# Supplementary material for: Factors Associated with the Abandonment of Exclusive Breastfeeding before Three Months
Source: Children (Basel). 2020 Dec 16;7(12):298. doi: 10.3390/children7120298 (PMC7766654; doi:10.3390/children7120298)
Supplement: Supplementary file 1 [file children-07-00298-s001.pdf]

Supplementary material S1: Baseline questionnaire

This survey is performed by the mother during the hospital stay after delivery.

Today's date:

MOHTER'S DATA:

1. Contact information:
    a) Mother's name and surname:
    b) Address:
    c) Email:
    d) Residence:
    e) Telephone numbers:
2. Age:
3. Nationality:
4. Marital status:
    a) Married
    b) Single
    c) Divorced
    d) Separated
    e) Widowed
    f) Single
5. Maternal education:
    a) Elementary
    b) Secondary school
    c) University
6. Date of birth of the newborn:
7. Number of previous children:
8. Number of previous abortions:
9. Parity: Primiparous   Multiparous
10. Do you live with a partner?:   Yes   No
11. Do you have help raising the infant?: Yes   No
12. Profession:
13. Employment situation:
    a) Unemployed
    b) Employed
    c) Housewife
    d) Maternity leave: Yes   No

DELIVERY AND INFANT DATA:

14. Hospital's name:
15. Multiple birth: Yes   No
16. Sex: Male   Female
17. Weight of newborn (in grams):

18. Mode delivery: Vaginal Caesarean section
19. Anaesthesia:
    a) Spinal
    b) Epidural
    c) General
    d) Local

BREASTFEEDING DATA:

20. Previous experience in breastfeeding (time in days)

Previous Children	Exclusive Breastfeeding	Mixed Breastfeeding	Formula Feeding
**1**			
**2**			
**3**			
**4**			
**5**			

21. What kind of lactation are you offering now?
    a) Exclusive breastfeeding
    b) Mixed breastfeeding
    c) Formula feeding
22. Do you have previous knowledge regarding breastfeeding? No Yes
23. If you have it, where did you acquired this knowledge?
    a) Journals/books
    b) Television
    c) Midwife
    d) Nurse
    e) Doctor
    f) Friends
    g) Family (indicate)
    h) Support group in health centre
    i) Previous experience
    j) Other (indicate)
24. Before childbirth, did you think about the kind of lactation you would offer your baby? Yes No
25. What type of lactation did you want to offer your baby?
    a) Exclusive breastfeeding
    b) Mixed breastfeeding
    c) Formula feeding
26. Now, what choice have you made?
    a) Exclusive breastfeeding
    b) Mixed breastfeeding
    c) Formula feeding
    d) I don't know

27. How long do you want to keep breastfeeding?
    a) 1 month
    b) 2 months
    c) 3 months
    d) Between 3 and 6 months
    e) As long as the baby wants it
    f) As long as I can to do it
28. Reason to start with formula bottles:
    a) Don't want to offer breastfeeding
    b) Work reasons
    c) Breastfeeding problems
    d) Influence of the family environment
    e) Influence of the health professional environment
29. Couple's opinion regarding breastfeeding:
    a) The couple is in agreement
    b) The couple is not in agreement
    c) Indifferent
30. Have you had problems with breastfeeding?: No Yes
31. Have health professionals helped you solve any problems?
    a) No
    b) Yes
    c) Not necessary
32. Did the baby receive a bottle of formula at the hospital? No Yes (indicate how much)
33. Has the baby received a bottle of water or oral rehydration serum? No Yes (indicate how much)
34. Do you think your baby gets hungry?
    a) No
    b) Yes
    c) I have doubts
35. Degree of satisfaction with health personnel:
    a) Wholly dissatisfied
    b) Not at all satisfied
    c) Moderately satisfied
    d) Highly satisfied

<u>Supplementary material S2: Questionnaire at 1,3 months (telephone survey)</u>

1. Mother's name and surname:
2. Date:
3. Medical record number of the mother:
4. Medical record number of the infant:

MOTHER'S DATA:

5. What kind of lactation are you offering now?
    a) Exclusive breastfeeding
    b) Mixed breastfeeding
    c) Formula feeding

6. Please tell us if you have had any health problems since the last interview and what this has been: Yes No

7. If you have had health problems, what did you do to fix it?
    a) I visited the doctor
    b) I visited the nurse/ midwife
    c) I asked people around me
    d) I solved it myself

8. Have you started to work? Yes, I have (indicate the date) No, I haven't

INFANT'S DATA:

9. Weight of newborn:
10. Does he/she use a dummy? Yes No
11. Does he/she been offered a bottle of formula? Yes No
12. If you have offered formula, how many bottles?
    a) Only one
    b) Every week
    c) Every day
